# Development, validity and reliability of the Italian version of the Copenhagen neck functional disability scale

**DOI:** 10.1186/s12891-018-2332-z

**Published:** 2018-11-23

**Authors:** Domenico Angilecchia, Maura Mezzetti, Alessandro Chiarotto, Antonella Daugenti, Giuseppe Giovannico, Francesca Bonetti

**Affiliations:** 1 Department of Physical Medicine and Rehabilitation OSMAIRM, via Cappuccini 9, 74014 Laterza, Taranto Italy; 2 University “Tor Vergata”, Faculty of Economics, Via Columbia, 2, 00133 Roma, Roma Italy; 3 Department of Epidemiolgy and Biostatistics, Amsterdam Movement Sciences research institute, VU University Medical Center, Amsterdam UMC, Amsterdam, Netherlands; 4 Department of General Practice, Erasmus Medical Center, Rotterdam, Netherlands; 5 Centro Giovanni Paolo II - Istituto Neuromed, Viale Europa, 70017 Putignano, Bari Italy; 6 Studio Professionale FTM, via Della Libertà 14, 73023 Lizzanello, Lecce Italy; 7 Physioup, Via Novacella 19, 00142 Roma, Roma Italy

**Keywords:** Chronic neck pain, Copenhagen neck functional disability scale, Outcome measure, Validity, Reliability, Italian

## Abstract

**Background:**

Valid and reliable patient-reported outcome measures support health professionals in evaluating the results of clinical research and practice. The Copenhagen Neck Functional Disability Scale (CNFDS) has shown promising measurement properties to measure disability in patients with neck pain, but an Italian version of this questionnaire is not available. The objective of this study was to cross-culturally adapt the CNFDS into Italian (CNFDS-I), and to assess its validity and reliability in patients with neck pain.

**Methods:**

The CNFDS-I was developed according to well-established guidelines for cross-cultural adaptation of patient-reported outcome measures. A cross-sectional clinimetric study was conducted to evaluate its validity and reliability. Patients with chronic neck pain (pain > 3 months) participated in this study. The following measurement properties (defined by the COSMIN initiative) were assessed: structural validity (exploratory factor analysis), internal consistency (Cronbach’s α), construct validity [by testing hypotheses on expected correlations with the Neck Disability Index (NDI), the Neck Bournemouth Questionnaire (NBQ), and pain Visual Analogue Scale (VAS)]. Test-retest reliability [Intraclass Correlation Coefficient for agreement (ICCagreement)], and measurement error [Smallest Detectable Change (SDC)] were also assessed in 50 clinically stable patients. Floor/ceiling effects and acceptability were calculated.

**Results:**

One-hundred and sixty-two patients (mean age = 47.9 ± 14.5 years, 70% female) were included. The CNFDS-I exhibited sufficient unidimensionality (one factor explained 83% of the variability) and internal consistency (α = 0.83). Construct validity was sufficient as all correlations with the other questionnaires were as expected (*r* = 0.846 with NDI, *r* = 0.708 with NBQ, *r* = 0.570 with VAS). Test-retest reliability was excellent (ICCagreement = 0.99, 95% CI from 0.995 to 0.999), while measurement error was equal to 8.31 scale points (27% scale range). No floor/ceiling effects were detected. The average time for filling the questionnaire was two minutes.

**Conclusions:**

The CNFDS-I proved to be a valid and reliable outcome measure to assess disability in patients with chronic neck pain. Head-to-head comparison studies on the CNFDS-I measurement properties against other disability measures for neck pain (e.g. NDI and NBQ) are required to determine the relative merits of these different measures.

**Electronic supplementary material:**

The online version of this article (10.1186/s12891-018-2332-z) contains supplementary material, which is available to authorized users.

## Background

Neck pain (NP) is a very prevalent musculoskeletal disorder worldwide; almost half of the population will experience a NP episode during the lifetime [1]. Most acute episodes of NP resolve with or without treatment, but almost 50% of people will continue to experience a certain degree of pain [2]. When symptoms associated to NP persists over three months, it is defined chronic NP [3]. A Neck Pain Task Force [4] has highlighted that about 10–20% of the European population displays chronic or persistent NP; it has also been shown that NP is the second cause of absence from work [5].

Neck pain is a multifactorial condition which can be related to sex, age, bad posture, poor state of health, other comorbidities, repetitive strain injuries, psychological factors, sleep disorder, and lifestyle [5, 6]. Therefore, patients with this condition present with a complex dysfunctional framework that clinicians should regularly manage the best possible manner. Within this context, it is useful to measure neck-related disability as perceived and described by the patient over time, irrespective of its etiology; this measurement can allow to monitor the patients’ outcomes, and to study the relationship between this outcome and other health-related and environmental factors [7].

The Copenhagen Neck Functional Disability Scale (CNFDS) was developed by Jordan et al., with the aim to make a new instrument to investigate the patient’s neck-related disability [8]; in contrast with other questionnaires (e.g. Neck Disability Index), it was not derived from already existing questionnaires [9, 10]. The functional disabilities experienced by patients with NP were used as a starting point by an interdisciplinary team of physiotherapists, rheumatologists and other professionals who aimed to develop this questionnaire as a self-reported tool, to avoid interviewer bias. The developers decided also not to include pain questions in the CNFDS, because these were considered to measure separate domains [11, 12]; their inclusion could also lead to problems with the unidimensionality of this questionnaire [8].The CNFDS has been shown to be strongly reliable and internally consistent, and to have excellent construct validity [8], similar to other instruments used to evaluate neck-related disability [13].

Considering that an Italian version of the CNFDS is not available for research and clinical purposes, this study aimed to cross-culturally adapt the CNFDS into the Italian language and culture, and to assess its measurement properties in patients with chronic NP. Making the CNFDS available in Italian will also allow to conduct head-to-head comparisons with other available neck-related disability questionnaires (e.g. Neck Disability Index [14], Neck Bournemouth Questionnaire [15]), to establish which one displays better measurement properties in NP patients.

## Materials and methods

This clinimetric study was approved by the ethics committee of the Brindisi ASL (Italy). All procedures were conducted according to the declaration of Helsinki, and all patients provided informed consent prior to study inclusion. Authorization to adapt the CNFDS into Italian was obtained from the original developer.

### Copenhagen neck functional disability scale

The CNFDS consists of 15 items that evaluate the impact of NP on headache, ability to sleep, concentration, activities related to work, daily activities and leisure activities. It includes also questions of psychosocial nature, such as decreased social contact, influence on emotional relationships with family members and attitudes toward the future. Each item can be answered as ‘yes’ (0 points), ‘occasionally’ (1 point), and ‘no (2 points). To avoid repetitive answering, response options are reversed after the fifth question. The total score can range from 0 to 30, with higher scores indicating worse disability.

### Translation and cross-cultural adaptation

The cross-cultural adaptation process was carried out in accordance with the recommendations proposed by Beaton et al. [16].

*Step 1: Forward translation to Italian*. Two Italian native speakers with good English knowledge independently translated the questionnaire into Italian. One translator was an economist with no medical background, the other was a speech therapist. Translators aimed at the conceptual equivalent of a word or phrase and used natural and acceptable language for the broadest audience range. The two translations were called T1 and T2.

*Step 2: Synthesis*. The two translators and four authors (DA, FB, AD, GG) discussed the translated questionnaire (T1 and T2) in a consensus meeting, to disentangle any discordant or ambiguous word and to develop a consensus-based version (T1–2).

*Step 3: Backward translation to English*. Two English native speakers without medical background, independently back-translated T1–2 into English. These translators were unfamiliar with the study purpose and were blinded to the original English version. Two back-translations were obtained (BT1 and BT2).

*Step 4: Expert committee*. The expert committee consisted of the four translators and four physiotherapists/authors (DA, FB, AD, GG). The committee reviewed all the translations (T1, T2, BT1, BT2) and compared the Italian version of the scale (T1–2) with the original version of the scale. Consensus in terms of semantic equivalence (ensuring that the words mean the same thing), idiomatic equivalence (formulation of equivalent expressions for colloquialisms), experiential equivalence (ensuring that each item properly captured the experience of daily life in target culture), and conceptual equivalence (ensuring that items hold the same conceptual meaning) was sought and achieved in the prefinal CNFDS version. The committee made only one change: in the first question “Can you sleep at night without neck pain interfering?” translated into Italian as “Riesce a dormire la notte senza che il dolore al collo possa disturbarla?” was changed into “Riesce a dormire la notte senza che il dolore al collo la disturbi?”

*Step 5: Pretesting*. The prefinal CNFDS was administered to 30 participants responding to the study inclusion criteria and who were asked about any misunderstanding, conflicting, or ambiguous word or sentence. These participants did not have any questions about the scale and all the questions were well understood. There was no multiple answer question or missing answer. After the pretesting, the final version of the scale was obtained (i.e. CNFDS-I), Additional file 1: Table S1.

### Participants

Consecutive outpatients seeking treatment or evaluation between March 2015 and February 2016 at the Physical Medicine and Rehabilitation Unit of “Organizzazione Sanitaria Meridionale Assistenza Inabili Recupero Minori” (OSMAIRM) were assessed for inclusion.

The inclusion criteria were: non-specific chronic NP (lasting > 12 wk) with or without arm pain, at least 18 years old, and ability to read and speak Italian fluently. The NP area was defined according to the IASP definition [17] as the area bounded by the nuchal fold on the top, an imaginary transverse line passing through the tip of the first chest spinous process on the bottom, and a sagittal suture tangential to the lateral edges of the neck on both sides.

Patients with neurological signs in the arms were excluded because there was a lack of instrumental examination for confirming the diagnosis (i.e. cervical radiculopathy) [18]. Other exclusion criteria were: lawsuits, cognitive impairment, fractures, cancer central neurological signs and severe psychiatric disorders.

### Other measurement instruments

A booklet asking information about demographic (e.g. age, sex) and clinical characteristics (e.g. pain duration) was administered to each patient. The same booklet contained the Italian versions of the Neck Disability Index (NDI-I) [14], of the Neck Bournemouth Questionnaire (NBQ-I) [15], and of the CNFDS-I; a Visual Analogue Scale (VAS) to assess pain intensity was also included [19].

The NDI is the most commonly tested and translated neck-related disability scale [20]. It consists of 10 items and each question is scored on a 6-point scale ranging from 0 (no disability) to 5 (full disability). Total score ranging from 0 to 50 which can also be expressed as a percentage. The NBQ is a short questionnaire consisting of seven items representing aspects of the biopsychosocial model relevant to patients with NP [21]. Each item is scored on a 0–10 numerical scale, where zero represents absence of limitation, for a total score ranging from 0 to 70 points. The NBQ-I has displayed acceptable construct validity and responsiveness in Italian patients with chronic NP [15].

The VAS is a horizontal line, 100 mm in length, asking patients to rate their pain intensity at the moment, “no pain” and “worst pain” are the extremes. The VAS has exhibited satisfactory test-retest reliability and construct validity in patients with chronic pain [22].

### Measurement analysis

The COnsensus-based standards for the Selection of health Measurement INstruments(COSMIN) definitions of measurement properties were adopted in this study [23].

#### Acceptability

The time needed to answer the questionnaire was recorded. Once completed, the patients were asked about any problems they encountered and the professionals administering the questionnaire inspected for the presence of missing or multiple responses.

#### Structural validity

Structural validity is the degree to which scores of an instrument adequately reflect the dimensionality of the construct to be measured [23]. Since this measurement property was not previously assessed for the CNFDS, an exploratory factor analysis was performed on the 15 items of the questionnaire, using a principal component estimator. The eigenvalue of each extracted factor was calculated, and a scree plot was drawn. A ratio between the first and the second eigenvalue larger than 4 was considered as an indication of unidimensionality [24]. Factor loadings for each item were also calculated, and loadings smaller than 0.4 were considered for item reduction of the questionnaire [25].

#### Internal consistency

This property reflects how much the items of a questionnaire are intercorrelated and it was assessed using Cronbach’s Alpha. The value is considered satisfactory if it is higher than 0.80 [26]. Correlation of each item with the total score of remaining items (item-total correlation) and inter-items correlation were also computed.

#### Construct validity

Construct validity was assessed by means of hypotheses testing, as suggested by the COSMIN initiative and the International Society for Quality of Life Research (ISOQOL) [27]. Hypotheses on expected correlations between CNFDS-I and the other questionnaires (i.e. NDI, NBQ and VAS) were tested. The correlation with NDI and NBQ was expected to be ≥0.60 as these questionnaires are considered to measure the similar construct (i.e. neck-related disability); the correlation with the VAS was expected to be ≥0.30 and < 0.60 as it measures a related (but not the same) construct. These hypotheses were in line with those formulated for similar and related constructs in a previous study in patients with NP [28]. Since data were normally distributed (i.e. Shapiro-Wilk test, *p*-value > 0.05), the correlations were assessed by means of Pearson’s correlation coefficient (rs). Construct validity was considered satisfactory if all three hypotheses were met [29].

#### Test-retest reliability

For the test-retest reliability, the questionnaire was administered twice to patients not receiving any treatment, with an interval period of 3 days. As suggested by Holt et al. [30], a long interval period may be inappropriate for a test-retest study of health measures because too many changes in the patient’s health status can occur. Before the second measurement, patients were asked if their NP condition had changed compared to the first measurement and only those reporting no change were re-assessed. The intraclass correlation coefficient (ICC) with a two-way mixed effect model for absolute agreement (ICCagreement) was calculated. An ICCagreement ≥0.70 indicated sufficient test-retest reliability [31].

#### Measurement error

Measurement error was assessed in stable patients that undertook the second measurement. The Standard Error of Measurement for agreement (SEM_agreement_) was computed by undertaking the square root of the within-people ANOVA sum of squares (i.e. sum of systematic and random error). The Smallest Detectable Change (SDC) was calculated with the following formula: SEM_agreement_ * 1.96 * √2. To judge whether a SDC is sufficiently small to allow measurement of a ‘real’ change, it should be compared with the minimal important change of an instrument [29]; however, since no minimal important change has been estimated for the CNFDS, this comparison was not feasible in this study.

#### Floor/ceiling effects

Floor and ceiling effects represented the percentage of patients with the minimal score (i.e. 0) and the percentage of patients with the maximum score (i.e. 30). Ceiling and floor effect were considered to be present if more than 15% of respondents achieved the lowest or highest possible total score [32].

## Results

Among the examined patients, 162 were eligible for this study. Fifty patients were (31%) clinically stable at the second measurement (after at least 3 days) and completed the CNFDS-I twice to assess reliability. The mean age of patients was 47,9 (standard deviation 14,5) years, most were female (69,7%), married (66,6%) and had completed secondary or university school (82,7%). Table 1 shows sociodemographic characteristics and it reports descriptive statistics for the total scores of CNFDS-I, NDI-I, NBQ-I and VAS. Descriptive statistics, item total correlations and the item rest correlations are illustrated for each CNFDS-I item in Table 2.

**Table 1:** Sociodemographic characteristics of the population (*n* = 162)

Variable	Value	*N*	%
Sex	F	113	69.8
	M	49	30.2
Married/Helpmate	Yes	108	66.7
	No	54	33.3
Education	Elementary	10	6.2
	Mid school	18	11.1
	High school	68	42.0
	Graduate school	66	40.7
Work	No (pain)	0	0
	Student	2	1.2
	Employee	84	51.9
	Self employed	30	18.5
	Retired	23	14.2
	Unemployed	3	1.9
	Housewife	20	12.3
Smoke	Yes	33	20.4
	No	103	63.6
	Ex smoker	26	16.0
Duration symptoms	From 3 to 6 months	48	29.6
	> 6 months	114	70.4
Physical activity or workout	No	85	52.5
	Yes < 3 h/week	45	27.8
	Yes > 3 h/week	32	19.8
Mean (SD) NDI-I	%	162	28.8 (14.7)
Mean (SD) NBQ-I	0–70	162	28,4 (14.2)
Mean (SD) VAS	0–100	162	45.5 (20.9)
Mean (SD) CNFDS-I	0–30	162	10.5 (5.9)
Mean (SD) CNFDS-I test-retest, 2nd test	0–30	50	10.92 (5.9)

*NDI* Neck Disability Index, *NBQ* Neck Bournemouth Questionnaire, *CNFDS* Copenhagen Neck Functional Disability Scale, *VAS* Visual Analogue Scale

**Table 2:** Descriptive Statistics, Factor Loadings, and item-total Correlations of the Items of the Questionnaire

Item	Description	Mean (SD)	Factor Loading	Item Total Correlation	Item rest Correlation
1	To sleep without pain	0.71 (0.77)	0.37	0.46	0.35
2	Daily activities levels	0.65 (0.70)	0.76	0.76	0.69
3	Daily activities without help from other	0.15 (0.49)	0.46	0.47	0.40
4	To manage clothes	0.21 (0.51)	0.49	0.52	0.45
5	To brush teeth	0.62 (0.75)	0.41	0.48	0.37
6	To spend time at home	0.63 (0.77)	0.73	0.73	0.66
7	To lift objects (2–4 kg)	0.92 (0.84)	0.46	0.52	0.41
8	To read	0.96 (0.83)	0.53	0.59	0.49
9	Headache	1.45 (0.71)	0.13	0.24	0.12
10	Ability to concentrate	0.97 (0.78)	0.52	0.58	0.48
11	Usual leisure/free time	0.88 (0.73)	0.61	0.62	0.53
12	To remain in bed	0.43 (0.68)	0.40	0.45	0.35
13	Emotional relationship	0.426 (0.694)	0.55	0.61	0.52
14	Social contacts	0.401 (0.682)	0.64	0.65	0.57
15	NP future influence	1.031 (0.814)	0.45	0.49	0.38

### Acceptability

All the questions were well accepted, there were no problems in the instrument’s comprehensibility. The questionnaire was completed with a mean of 120 s (standard deviation = 40). No missing responses or multiple answers were found.

### Structural validity

**Fig. 1:**
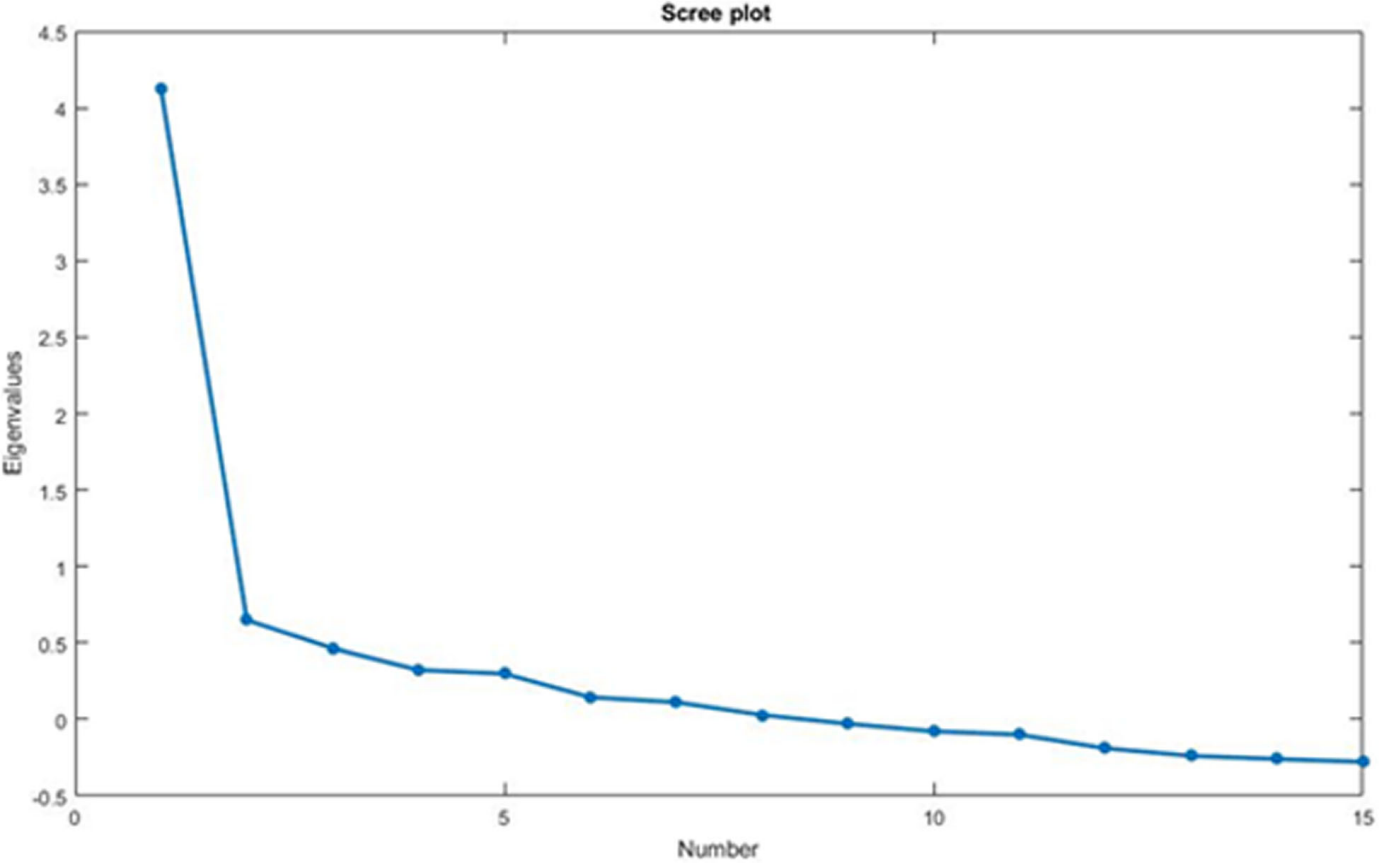
Scree plot of eigenvalues after factor analysis. The scree plot displays the number of the factor versus its corresponding eigenvalue. It shows that the first factor explained 83% of the total variability of data, while the second factor explained only 13%

Exploratory factor analysis revealed that the first factor explained 83% of the total variability (eigenvalue = 4.122), while the second factor explained only 13% (eigenvalue = 0.648). The ratio between the first and the second eigenvalues was equal to 6.36. The scree plot (Fig. 1) also indicated a clear unidimensional pattern for the CNFDS-I. Factor loadings are presented in Table 2 and show as the item 9 appear deflected. We decided not to delete this item because we consider its content (Have you been bothered by headaches during the time that you have had neck pain?) very relevant to patients with NP, because it presented a sufficient item total correlation (Table 2), and because we would not like to create a CNFDS Italian version that differs from other versions in the number of items.

### Internal consistency

The Cronbach α for the scale was 0.83, indicating satisfactory results. Cronbach’s α when an item was excluded ranged from 0.12 to 0.69, while correlation of each item with the total score ranged from 0.24 to 0.76 (Table 2).

### Construct validity

CNFDS-I correlations with the NDI-I and the NBQ-I were equal to 0.846 and 0.708, respectively. The correlation between the CNFDS-I and the VAS was 0.570. All these correlations met the expected hypotheses and the CNFDS-I construct validity was considered satisfactory.

Summary in Table 3.

**Table 3:** Comparison Measurement Properties of CNFDS Adaptations and Construct Validity

	Internal Consistency (α)	Test-Retest Reliability (ICC)	SEM - SDC	Structural Validity (explained variance)	Costruct Validity (correlation)
CNFDS (Jordan A et al.)	0.90	0.99 (same day) - 0.90 (by mail 2 days later)	/ - /	/	*r* = 0.83 with pain score *r* = 0.89 with patient global assessment *r* = 0.56 doctor global assessment
CNFDS France (Forestier R et al.)	0.83	/	/ - /	/	*r* = 0.45 with VAS
CNFDS Poland (Misterska F et al.)	0.90	0.93 (24 h)	/ - /	/	*r* = 0.87 with NDI
CNFDS Turkey (Yapali G et al.)	/	0.86 (7 days)	/ - /	/	*r* = 0.78 with NPDS *r* = 0.73 with VAS
CNFDS Iran (Nayeb Aghaei H et al.)	0.84	0.95 (n.d.)	/ - /	/	*r* > 0.4 between each item and the three CNFDS subscales *r* = 0.80 with mJOA
CNFDS Italy	0.83	0.99 (3 days)	3 points - 8.31 points	6.36	*r* = 0.85 with NDI-I *r* = 0.71 with NBQ-I *r* = 0.57 with VAS

*CNFDS* Copenhagen Neck Functional Disability Scale, *SEM* Standard Error of Measurement, *SDC* Smallest Detectable Change, *VAS* Visual Analogue Scale, *NPDS* Neck Pain and Disability Scale, *mJOA* modified Japanese Orthopedic Association, *NDI* Neck Disability Index, *NBQ* Neck Bournemouth Questionnaire

### Test-retest reliability

The ICC_agreement_ was equal to 0.997 with a 95% confidence interval ranging from 0.995 to 0.999. This result represents excellent test-retest reliability.

### Measurement error

The SEM_agreement_ equalled 3.0 scale points (10% of the scale range), while the SDC was 8.31 scale points (27% of the scale range).

### Floor/ceiling effects

The CNFDS-I had no floor or ceiling effects, in fact no patients with a minimum and maximum score were identified in the test nor in the retest.

## Discussion

### Summary of the study

This study describes the cross-cultural adaptation and evaluation of the CNFDS-I in patients with chronic NP. The CNFDS-I is a unidimensional and internally consistent tool with excellent test-retest reliability and sufficient construct validity; the SDC was equal to 8.31 scale points (27% of the scale range). These results indicate that the CNFDS-I is ready to be used as a measuring method of neck-related disability in Italian patients with chronic NP.

This is the first study to perform a factor analysis of the CNFDS as the original study [8], while the previous ones [33–36] did not do that. The CNFDS-I resulted to be unidimensional and this represents a key finding, as it indicates that it is appropriate to use its total sum score [24]. Meanwhile, this finding does not support the use of the subscales proposed by the French developers [35]. In contrast with the CNFDS-I, other neck-related disability tools available in Italian (i.e. NDI-I and NPQ-I) were not shown to be unidimensional [14, 15, 37], questioning the suitability of using their total sum scores.

### Strengths and limitations

The unidimensionality of the NDI has been questioned also by other studies in other language versions [38, 39]. A future study can aim at comparing directly the unidimensionality of all these tools, to check if the CNFDS is indeed the best performing method from a psychometric point of view. Item 9 was the only CNFDS-I item with a low factor loading; future studies should assess if this result is repeated in other languages.

The Cronbach’s α of the entire questionnaire (α = 0.83) was major than the predefined threshold, and it was similar to the values obtained in the original version (α = 0.90) French (α = 0.83) Polish (α = 0.90) and Iranian (α = 0.84) [33–36]. The comparison is simplified in Table 3. This indicates a high interrelation of the items.

Construct validity was analyzed by comparing the CNFDS-I to the NDI-I and NBQ-I. The very high correlation between CNFDS-I and NDI-I (ρ = 0.846) suggests that the theoretical construct of these two instruments may be very similar [32]. We chose the NDI as a comparative questionnaire because it is the most validated and internationally used [15, 40]. The correlation between the total scores of CNFDS-I and NBQ-I was high too (ρ = 0.708). A second disability comparator questionnaire was included to compare the biopsychosocial aspects examined by the NBQ-I [13, 21]; the correlation between the two questionnaires outlines that their content is not totally different as described by Ferreira et al. [13], possibly because they may differ across cultures. Foremost, to fully compare the content validity of these different instruments, it is necessary to conduct a head-to-head comparison study asking patients and clinicians if the most relevant neck-related disability aspects are included in each questionnaire [8]. Additionally, a consensus-based definition for neck-related disability should be established before further content assessment of the tools. These considerations are not totally new, as a recent systematic review clearly highlighted that the content validity of patient-reported outcome measures is understudied [41], and that a major effort should be made to fill this evidence gap.

The authors of French version [35], compared the CNFDS with the VAS scale, found a moderate correlation (0,45 Spearman’s r).

Similarly, we have identified a correlation of ρ = 0.570. This result reflects those by Fejer [42], highlighting the moderate correlation between NP intensity and disability, which are strongly associated. Therefore, the CNFDS and the VAS evaluate different aspects, though maintaining a certain degree of correlation.

Moreover, after validating the CNFDS in Iran, Azhari et al. [43] investigated the most important aspects covered by the CNFDS, emphasizing the fact that its primary use is to gather and measure the patients’ disability, not their perceived pain.

CNFDS-I test-retest reliability was excellent as highlighted in the original development study [8] and other studies [24, 34–36]. The ICC agreement represents an absolute measure of reliability and it is suggested that it should be interpreted in combination with a relative measure such as the SDC [44]. The SDC of the CNFDS-I was found to be 27% of the scale range; this may suggest that repeated measurements with this tool do not give similar results, but there is not a standard cut-off value to determine whether a SDS is indeed small enough. Moreover, a measurement error larger than 20% of the scale range has been found for various broadly used questionnaires [45]. A comparison between SDC and minimal important change should be performed to have a better insight into the CNFDS measurement error. In light of these considerations, future studies on the CNFDS should calculate its minimal important change to be able to better interpret this instruments’ changes in scores.

### Unanswered questions and future research

This study did not assess responsiveness, defined as the ability of an instrument to detect changes in the construct to be measured [23]. It is an important measurement property to use an instrument as an outcome measurement instrument and it has not been evaluated in any language for the CNFDS. Overall, it remains unclear which neck-related disability instrument should be preferred in Italian patients with NP; thus, there is an urgent need of a head-to-head clinimetric study comparing content validity, structural validity and responsiveness of various instruments (including NDI-I and NBQ-I) in the same patients.

## Conclusion

To sum up, the CNFDS-I was found to be a unidimensional, valid and reliable tool in patients with chronic NP. In absence of comparative evidence showing that the neck-related disability instrument is superior from a measurement point of view, the CNFDS-I can be used alongside other more widely used tools (e.g. NDI and NBQ), for research and routine clinical monitoring in patients with chronic NP.

## Additional files

Additional file 1: **Table S1.** CNFDS-I Copenhagen Neck Functional Disability Scale. The English questionnaire “Copenhagen Neck Functional Disability Scale” and the Italian version. It is reliable and valid evaluation instrument for disability in patients with neck pain. (DOCX 18 kb)

## Abbreviations

CNFDS: Copenhagen neck functional disability scale
CNFDS-I: Copenhagen neck functional disability scale – Italian version
COSMIN: COnsensus-based standards for the selection of health measurement INstruments
ICC: Intraclass correlation coefficient
NBQ: Neck bournemouth questionnaire
NBQ-I: Neck Bournemouth questionnaire – Italian version
NDI: Neck disability index
NDI-I: Neck disability index – Italian version
NP: Neck pain
SDC: Smallest detectable change
SEM: Standard error of measurement
VAS: Visual analogue scale
