# Supplementary material for: Development, validity and reliability of the Italian version of the Copenhagen neck functional disability scale
Source: BMC Musculoskelet Disord. 2018 Nov 23;19:409. doi: 10.1186/s12891-018-2332-z (PMC6260862; doi:10.1186/s12891-018-2332-z)
Supplement: Supplementary file 1 — Table S1. CNFDS-I Copenhagen Neck Functional Disability Scale. The English questionnaire “Copenhagen Neck Functional Disability Scale” and the Italian version. It is reliable and valid evaluation instrument for disability in patients with neck pain. (DOCX 18 kb) [file 12891_2018_2332_MOESM1_ESM.docx]

**Copenhagen neck functional disability scale**

Si prega di indicare quanto sia stato interessato dal dolore al collo durante l’ultima settimana (o in un altro periodo concordato), barrando la casella appropriata nelle colonne alla destra di ciascuna domanda:

*Angilecchia D, Mezzetti M, Chiarotto A, Daugenti A, Giovannico G, Bonetti F*.

|  |  |  |  | - |
| --- | --- | --- | --- | --- |
|  |  | ***SI*** | ***OCCASIONALMENTE*** | ***NO*** |
| ***1*** | Riesce a dormire la notte senza che il dolore al collo la disturbi? |  |  |  |
| ***2*** | Riesce a svolgere le attività quotidiane senza che il dolore al collo riduca i livelli di attività? |  |  |  |
| ***3*** | Riesce a svolgere le attività quotidiane senza aiuto da parte di altri? |  |  |  |
| ***4*** | Riesce a indossare i vestiti al mattino, senza impiegare più tempo del solito? |  |  |  |
| ***5*** | Riesce a piegarsi sul lavandino per lavare i denti senza che le venga dolore al collo? |  |  |  |
| ***6*** | Trascorre più tempo del solito a casa, a causa del dolore al collo? |  |  |  |
| ***7*** | Evita di sollevare oggetti che pesano dai 2 ai 4 kg, a causa del dolore al collo? |  |  |  |
| ***8*** | Ha ridotto il tempo che trascorre a leggere a causa del dolore al collo? |  |  |  |
| ***9*** | E’ stato infastidito da mal di testa durante il periodo in cui ha avuto dolore al collo? |  |  |  |
| ***10*** | Pensa che la sua capacità di concentrazione si sia ridotta a causa del dolore al collo? |  |  |  |
| ***11*** | E’ ostacolato nello svolgere le sue abituali attività di tempo libero a causa del dolore al collo? |  |  |  |
| ***12*** | Rimane a letto più a lungo del solito a causa del dolore al collo? |  |  |  |
| ***13*** | Pensa che il dolore al collo abbia influenzato le sue relazioni affettive con i familiari più vicini? |  |  |  |
| ***14*** | Ha dovuto rinunciare alle relazioni sociali durante le ultime due settimane, a causa del dolore al collo? |  |  |  |
| ***15*** | Pensa che il suo dolore al collo possa influenzare il suo futuro? |  |  |  |
|  |  |  |  |  |
|  |  |  |  |  |

Nome___________________________ Cognome_______________________ Data__/__/____

**Copenhagen neck functional disability scale**

Please indicate how your neck pain has been affecting you during the last week or other agreed time period by circling the appropriate number in the columns to the right of each question:

|  |  |  |  | - |
| --- | --- | --- | --- | --- |
|  |  | ***yes*** | ***occasionally*** | ***no*** |
| ***1*** | can you sleep at night without neck pain interfering? |  |  |  |
| ***2*** | can you manage daily activities without neck pain reducing activity levels? |  |  |  |
| ***3*** | can you manage daily activities without help from others? |  |  |  |
| ***4*** | can you manage putting on your clothes in the morning without taking more time than usual? |  |  |  |
| ***5*** | can you bend over the wash basin in order to brush your teeth without getting neck pain? |  |  |  |
| ***6*** | do you spend more time than usual at home because of neck pain? |  |  |  |
| ***7*** | are you prevented from lifting objects weighing from 2-4 kg. due to neck pain? |  |  |  |
| ***8*** | have you reduced your reading activity due to neck pain? |  |  |  |
| ***9*** | have you been bothered by headaches during the time that you have had neck pain? |  |  |  |
| ***10*** | do you feel that your ability to concentrate is reduced due to neck pain? |  |  |  |
| ***11*** | are you prevented from participating in your usual leisure time activities due to neck pain? |  |  |  |
| ***12*** | do you remain in bed longer than usual due to neck pain? |  |  |  |
| ***13*** | do you feel that neck pain has influenced your emotional relationship with your nearest family? |  |  |  |
| ***14*** | have you had to give up social contact with other people during the past two weeks due to neck pain? |  |  |  |
| ***15*** | do you feel that neck pain will influence your future? |  |  |  |
|  |  |  |  |  |
